# Mechanical Control of the Optical Bandgap in One-Dimensional Photonic Crystals

**DOI:** 10.3390/mi13122248

**Published:** 2022-12-17

**Authors:** V. Paige Stinson, Nuren Shuchi, Micheal McLamb, Glenn D. Boreman, Tino Hofmann

**Affiliations:** Department of Physics and Optical Science, University of North Carolina at Charlotte, 9201 University City Blvd, Charlotte, NC 28223, USA

**Keywords:** photonic crystal, two-photon polymerization, photonic bandgap, mechanical flexure, opto-mechanics

## Abstract

Over the last several years, two-photon polymerization has been a popular fabrication approach for photonic crystals due to its high spatial resolution. One-dimensional photonic crystals with photonic bandgap reflectivities over 90% have been demonstrated for the infrared spectral range. With the success of these structures, methods which can provide tunability of the photonic bandgap are being explored. In this study, we demonstrate the use of mechanical flexures in the design of one-dimensional photonic crystals fabricated by two-photon polymerization for the first time. Experimental results show that these photonic crystals provide active mechanically induced spectral control of the photonic bandgap. An analysis of the mechanical behavior of the photonic crystal is presented and elastic behavior is observed. These results suggest that one-dimensional photonic crystals with mechanical flexures can successfully function as opto-mechanical structures.

## 1. Introduction

Photonic crystals have been explored for photonic bandgap filtering applications over the last several decades [1,2,3,4,5,6]. This is due to their ability to provide nearly perfect reflection within narrow bandgaps which can function over a broad spectral range. These spectral regions with high reflectivities and little to no transmission are known as photonic bandgaps. Photonic crystals induce a photonic bandgap by creating a dielectric periodicity in either one-, two-, or three-dimensions [5]. In the one-dimensional case, this periodicity is created along a single axis [1,2,7,8,9,10,11,12].

There are several effective fabrication approaches for one-dimensional photonic crystals such as spin coating, self-assembly, chemical/physical vapor deposition, top-down etching, and molecular beam epitaxy [13,14]. While these approaches each have their advantages, they are restricted in terms of geometrical design freedom. A recent approach in the fabrication of one-dimensional photonic crystals is direct laser writing by two-photon polymerization [11,15,16,17]. This approach allows for the fabrication of complex geometrical structures on a scale which is sub-wavelength for most infrared applications [18,19]. With the aforementioned fabrication approaches, at least two dielectric materials must be used in order to form the necessary dielectric periodicity. In direct laser writing, dielectric periodicity can be produced using a single dielectric material by varying the density of the layers [11,17].

While the highly reflective photonic bandgaps are in themselves extremely useful spectral features, many researchers have looked to provide even further spectral control by mechanical means [6,12,20,21]. Many of these studies explore the ability to control spectral features produced by photonic crystals by mechanically straining the material [20,21,22]. For the one-dimensional photonic crystal, the photonic bandgap is extremely sensitive to changes in layer thickness. By introducing layers containing mechanically flexible constituents [23,24] a mechanical force can be used to either expand or compress the photonic crystal. This will allow the active control of the photonic bandgap by mechanical stimuli. This concept has been demonstrated in the THz spectral range by implementing cantilevers into the one-dimensional photonic crystal design [23].

In this study, we present a one-dimensional photonic crystal design which allows mechanically induced spectral tuning of the photonic bandgap in the infrared spectral range for the first time. To accomplish this, a bow tie flexure design adapted from reference [24] is scaled down such that the dimensions are subwavelength for the spectral range of interest in this study. Experimental results suggest that these one-dimensional photonic crystals with flexures fabricated by two-photon polymerization exhibit mechanical hysteresis while in compression, as expected of an elastic material [25]. The results reported here suggest that one-dimensional photonic crystals with flexures fabricated by two-photon polymerization may be used in novel opto-mechanical devices enabling mechanical tuning of optical features.

## 2. Design, Fabrication, and Characterization

**Figure 1:**
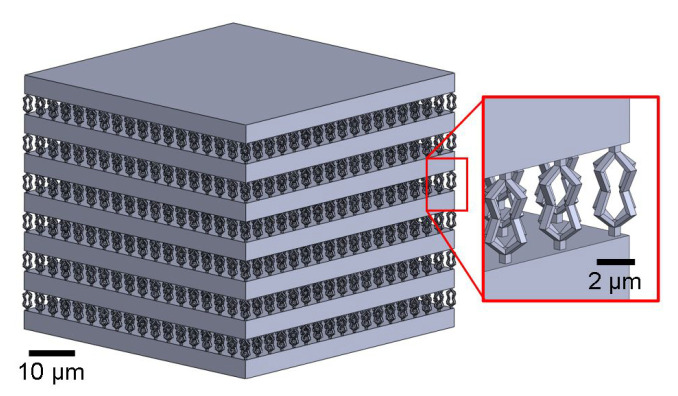
Computer-aided model of the designed one-dimensional photonic crystal investigated here. The photonic crystal consists of alternating high-density compact and low-density layers. The nominal thickness of the high-density layers is 3.35 µm. The low-density layers with a nominal thickness of 3.40 µm are composed of an array of bow tie flexures which are arranged in a square-lattice pattern on the surface with a lattice constant of 2.85 µm. The corresponding nominal volumetric fill factor $f_{i}$ of the low-density layer is $f_{i}=0.04$.

The one-dimensional photonic crystals under study were designed using a stratified-layer optical model (WVASE32, J.A. Woollam, Co., Lincoln, NE, USA) in combination with a mechanical finite-element model (COMSOL, Multiphysics). A single photo-sensitive polymer (IP-Dip) was used to fabricate the one-dimensional photonic crystal. The mechanical properties for IP-Dip vary widely depending on fabrication parameters, the properties which were employed during finite-element modeling were obtained from references [26,27]. To induce dielectric periodicity using a single polymer, 13 plane parallel layers of alternating high- and low-density were used (see Figure 1). The dielectric properties of the compact, high-density, layers have been previously established using spectroscopic ellipsometry in the infrared spectral range and are described using a simple mixed oscillator model [28]. The low-density layers consist of the printed mechanical flexures and the void (air) between adjacent flexures. The dielectric properties of these layers are calculated using the Bruggeman effective medium approximation [29]. The use of this approximation to model the spectral response of one-dimensional photonic crystals is described in detail in previous publications [7,11,17,23,30].

To demonstrate the ability of the designed photonic crystals to produce spectral bandgap shifts upon compression, the layer thickness and dielectric composition of the low-density layers were designed such that several bandgaps were induced within the measurement window (1000–5000 cm${}^{-1}$). Within this region, the initial bandgap is induced at 2000 cm${}^{-1}$ and the final at 4700 cm${}^{-1}$. This spectral range was selected based on the dielectric function of IP-Dip which offers a transparent window from 1800 to 5000 cm${}^{-1}$ [28]. The high-density layer thickness was designed to be 3.35 µm. The low-density layer thickness was designed to be 3.40 µm. Each low-density layer consists of an 18 × 18 array of bow tie flexures, arranged in a square lattice pattern with a periodicity of 2.85 µm. The base of the one-dimensional photonic crystal is 49.2 µm × 49.2 µm. The computer-aided design for the resulting geometry can be seen in Figure 1.

A commercially available two-photon polymerization system (Photonic Professional GT, Nanoscribe, GmBH) was employed in the fabrication of these one-dimensional photonic crystals from a single photosensitive monomer (IP-Dip). The selection of appropriate print parameters is essential in order to produce a sample which is true to design. The designed bow tie flexures push the resolution limits of this system, limiting the range of print parameters which can successfully produce a given geometry. Many print parameters such as the choice of objective, monomer, laser power, scan speed, and hatching and slicing distance will affect the voxel size of the system [31,32,33,34]. The voxel is the ellipsoidal volume in which there is enough photon irradiance to polymerize the monomer. To efficiently determine the best settings for this geometry, a dose matrix was performed in which several combinations of scan speed and laser power were tested. Here the 63x objective was chosen with both hatching and slicing distances set to 0.2 µm. A single low-density layer was printed on top of a high-density layer. Scanning electron microscope images were taken in order to compare the quality. Scan speeds ranging from 500 to 5000 mm/s were tested with laser powers ranging from 20 to 50% of the maximum output power of 25 kW.

**Figure 2:**
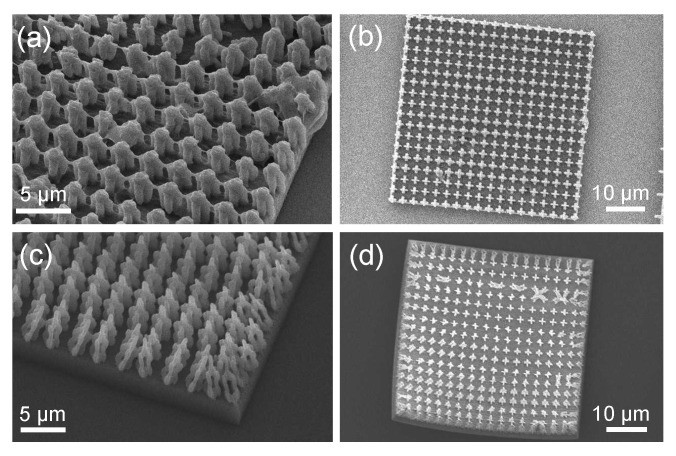
Scanning electron microscope images of single flexure layers which were fabricated with different laser power settings. Images (**a**,**b**) show two views of a flexure layer which was fabricated using 500 mm/s scan speed with 50% laser power. Images (**c**,**d**) show the same views of a layer fabricated using 500 mm/s scan speed with 20% laser power.

The scanning electron microscope images for the two best scan speed and laser power combinations can be seen in Figure 2. From the top down view given by (b) and (d), it appears that the 50% laser power resulted in the best geometry compared with the 20% layer. However, upon taking a side view of these layers at a higher magnification it can be seen that the layer fabricated at 50% laser power (a) has been over-polymerized compared to the layer fabricated at 20% laser power (c). In order to ensure a hollow center within the flexures, a lower laser power must be used. There is an apparent compromise in structural stability as laser power is decreased, which can be seen in the collapse of flexures in (d) compared to the stable flexures in (b). However, when fabricating the complete one-dimensional photonic crystal, this behavior is not an issue because the high-density layers provide the necessary permanence to maintain an upright orientation. Thus, a scan speed of 500 mm/s and laser power of 20% was chosen for the fabrication of the complete photonic crystal.

A 4 × 4 array of one-dimensional photonic crystals with 3 mm square lattice periodicity were fabricated on a fused silica substrate. This arrangement was chosen based on finite-element model calculations of the elastic properties of the bow tie flexure design. Compressive force was applied to the photonic crystals in increments. In order to provide an even distribution of pressure while maintaining transparency for measurement, a fused silica window with thickness 0.7 mm was placed on the array. This window was measured to have a mass of 1.37 g. Subsequent slides were placed on top of the initial window in log-cabin style to provide incremental increases in compressive force without interfering with the central measurement aperture. These subsequent increases in loading were done in steps of 2.83 g.

Infrared reflection measurements were taken of the photonic crystal for the spectral range from 1000 cm${}^{-1}$ to 5000 cm${}^{-1}$ with resolution 2 cm${}^{-1}$. An IR microscope (HYPERION 3000 Bruker, Inc., Billerica, MA, USA) with a Fourier-transform infrared spectrometer (VERTEX 70, Bruker, Inc., Billerica, MA, USA) was used for these measurements. This system uses a silicon carbide globar as the infrared light source. A 15× cassegrain objective with a square aperture of 20 µm × 20 µm was selected, resulting in an angular spread of 0.6° with an average angle of incidence of 8.7°. All measurements were normalized to the reflection of a bulk gold sample using identical parameters to those aforementioned. Experimental measurements were taken at room temperature.

## 3. Results and Discussion

**Figure 3:**
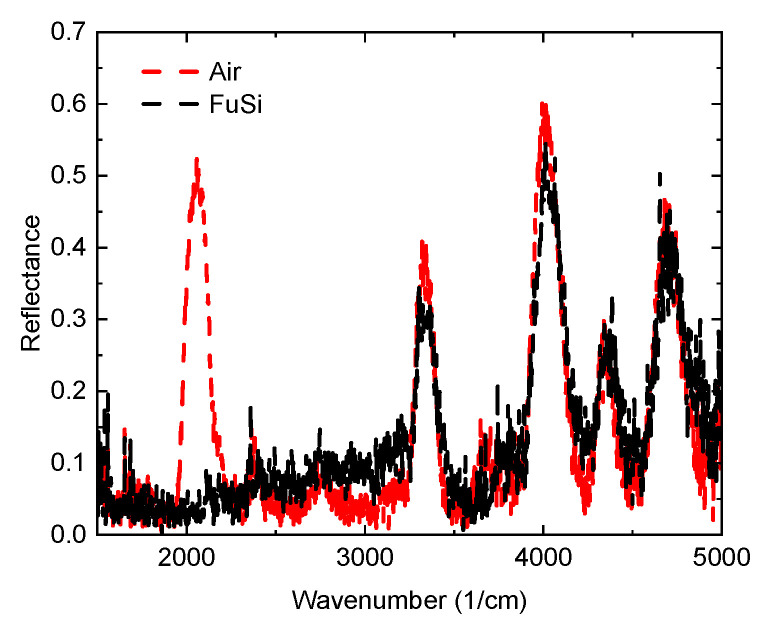
Fourier-transform infrared reflection measurements of the fabricated photonic crystal measured both in air (black dashed) and through a Fused Silica substrate (red dashed). At this stage, there is no compression on the photonic crystal.

In order to determine which photonic bandgaps were appropriate to observe upon compression, two Fourier-transform infrared reflection measurements were taken of the photonic crystal both with and without the presence of the fused silica window. Here kapton tape spacers are placed around around the edges of the fused silica window in order to prevent compression, allowing the effects of the fused silica window on the photonic bandgaps to be isolated. These measurements are given in Figure 3. The red dashed curve shows the measurements for the photonic crystal in air where four distinct photonic bandgaps can be seen. The black dashed curve shows the measurements where the fused silica window has been introduced but is not yet in contact with the structures. From this comparison, it is apparent that three of the four photonic bandgaps induced within this spectral range experience minimal amplitude loss due to the fused silica window. Thus, the photonic bandgap centered at 4040 cm${}^{-1}$ was chosen to observe due to its amplitude and position within the measurement window.

**Figure 4:**
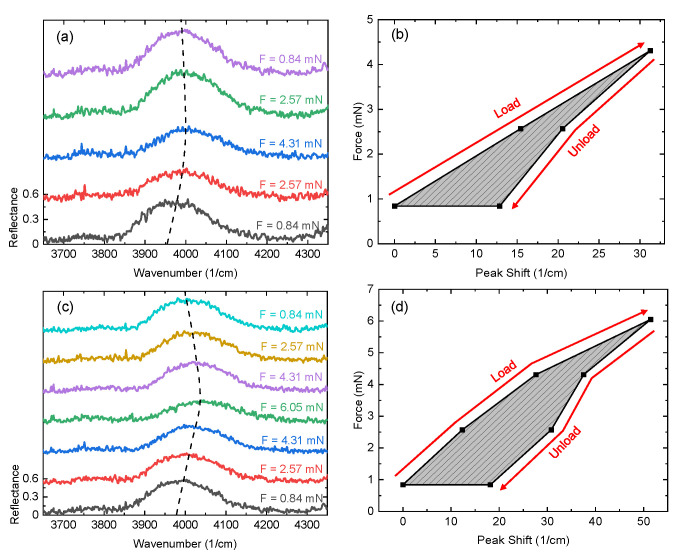
Effect of compressive force on photonic bandgap spectral positioning and amplitude for two cycles. Here the photonic bandgap centered at 4040 cm${}^{-1}$ in Figure 3 is isolated and studied. The Fourier-transform infrared reflection measurements for this bandgap are given in (**a**,**c**) for loading and unloading where a shift of the peak center can be seen as well as fluctuations in amplitude. An offset of 0.5 relative reflection intensity is introduced between adjacent curves for visualization. A mechanical hysteresis curve following the compressive cycles of (**a**,**c**) is plotted in (**b**,**d**), respectively. Here compressive force is plotted as a function of shift in peak center.

The experimental reflection data for two compressive cycles are given in Figure 4 where the measurement window is centered around the 4040 cm${}^{-1}$ bandgap. For the first point in the compressive cycle shown in Figure 4b,d, the peak shift is 0 cm${}^{-1}$. The reflection spectra for these initial points in the load curve can be seen in Figure 4a,c, respectively, for F = 0.84 mN. For each point in (b), as the photonic crystal is loaded and unloaded, the peak center is measured from (a) and the peak shift is recorded in (b) as a function of compressive force. The same process is followed for the second cycle given in (c) and (d). For visual clarity, each subsequent curve in (a) is vertically offset by 0.5 relative reflection intensity. During these cycles, the rate of shift is higher during loading than unloading. Comparing the photonic bandgaps during this cycle in (a) it is clear the peak center does not return to the initial position even though it is under the same compressive force. During the second compressive cycle, the maximum compressive force is increased from 4.31 mN per photonic crystal to 6.05 mN. While the degree of shifting varies between cycles, the general shape of the curves is similar (see Figure 4b,d). Peak shifting to larger wavenumbers during loading aligns with the expected effects of reducing the low-density layer thickness. Similarly, shifting to smaller wavenumbers during unloading is expected as low-density layer thickness is increased.

## 4. Conclusions

One-dimensional photonic crystals with mechanical flexures fabricated by two-photon polymerization have demonstrated spectral shifting as a function of compressive force. The photonic crystal consists of 13 alternating layers of high- and low-density. The ability of such a device to induce high-contrast photonic bandgaps in the infrared spectral range has been demonstrated previously [11,17]. In this study, mechanical flexure arrays are implemented to function as the low-density layers. Due to the photonic bandgap’s sensitivity to changes in layer thickness, compression of the photonic crystal induces spectral shifting of the photonic bandgaps which can be observed in Figure 4. Such an effect has been previously demonstrated in the THz spectral range using stereo-lithography [23]. In contrast to the previous THz demonstration, the low-density layer geometry was altered in this study to include vertically symmetric flexure arrays as opposed to cantilevers arrays. When comparing compressive effects on the photonic bandgap observed in this study with the THz demonstration, it appears that the effects on bandgap amplitude and broadening during compression are reduced.

In conclusion, a mechanically tunable one-dimensional photonic crystal designed for the infrared spectral range was fabricated using two-photon polymerization. The elastic capabilities of this device were tested by conducting loading and unloading cycles. During cycles, Fourier-transform infrared reflection measurements were taken to observe the effect of compressive force on the photonic bandgap’s spectral location and amplitude. The results of this study suggest one-dimensional photonic crystals with mechanical flexures allow dynamic control of spectral features by way of mechanical stimuli. While static one-dimensional photonic crystals have clear applications in sensing and bandgap filtering, the addition of mechanical tunability opens doors for uses in fields such as micro-robotics and micro-optomechanical systems (MOEMS) [35].

## Data Availability

Not applicable.
